# Fibroblast Growth Factor Receptor 4 Targeting in Cancer: New Insights into Mechanisms and Therapeutic Strategies †

**DOI:** 10.3390/cells8010031

**Published:** 2019-01-09

**Authors:** Liwei Lang, Yong Teng

**Affiliations:** 1 Department of Oral Biology and Diagnostic Sciences, Dental College of Georgia, Augusta University, Augusta, GA 30912, USA; llang@augusta.edu; 2 Georgia Cancer Center, Department of Biochemistry and Molecular Biology, Medical College of Georgia, Augusta University, Augusta, GA 30912, USA; 3 Department of Medical Laboratory, Imaging and Radiologic Sciences, College of Allied Health, Augusta University, Augusta, GA 30912, USA

**Keywords:** FGFR4, FGF19, gene regulation, cancer signaling, anticancer

## Abstract

Fibroblast growth factor receptor 4 (FGFR4), a tyrosine kinase receptor for FGFs, is involved in diverse cellular processes, including the regulation of cell proliferation, differentiation, migration, metabolism, and bile acid biosynthesis. High activation of FGFR4 is strongly associated with the amplification of its specific ligand FGF19 in many types of solid tumors and hematologic malignancies, where it acts as an oncogene driving the cancer development and progression. Currently, the development and therapeutic evaluation of FGFR4-specific inhibitors, such as BLU9931 and H3B-6527, in animal models and cancer patients, are paving the way to suppress hyperactive FGFR4 signaling in cancer. This comprehensive review not only covers the recent discoveries in understanding FGFR4 regulation and function in cancer, but also reveals the therapeutic implications and applications regarding emerging anti-FGFR4 agents. Our aim is to pinpoint the potential of FGFR4 as a therapeutic target and identify new avenues for advancing future research in the field.

## 1. Introduction

Fibroblast growth factor receptors (FGFRs) have been found to play a vital role in tumorigenesis and cancer progression through increased cell proliferation, metastasis, and survival [1,2]. Compared with the other three FGFR family members, the signaling pathways and mechanisms of FGFR4 involved in cancer development are less characterized. The expression of FGFR4 is strictly regulated in human adult organs and tissues after fetal development, suggesting it perhaps has a particular relevance to tissue functions. Recently, elevated FGFR4 has been tightly correlated with cancer development and progression, making it an attractive target to develop novel and effective anticancer therapeutics. More efforts have been focused on developing selective inhibitors to target FGFR4, which show particular promise as an anticancer monotherapy or an adjunct treatment.

## 2. Molecular Characters of FGFR4 and Its Ligands

### 2.1. The Molecular Structure of FGFR4

**Figure 1:**
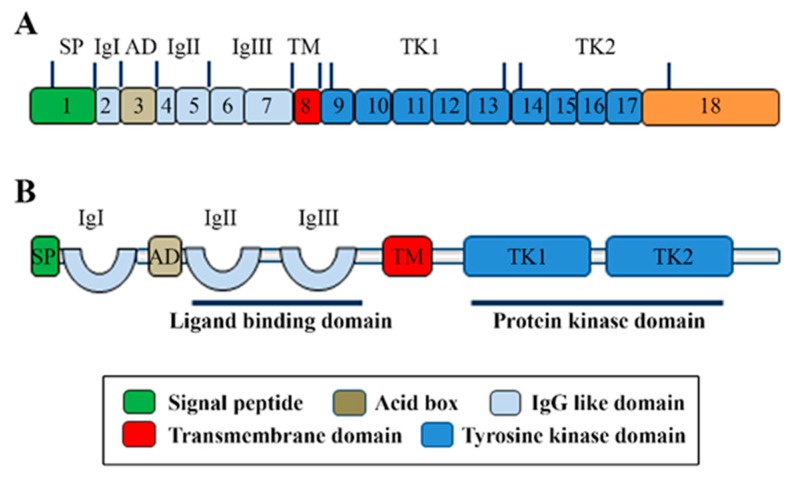
The molecular structure of FGFR4. (**A**) The illustration of FGFR4 with mRNA structure. The transcript variant 1 of FGFR4 contains 18 exons and encodes isoform 1 of FGFR4 protein with the main function domains. (**B**) The main domains of FGFR4 with the corresponding function.

**Figure 2:**
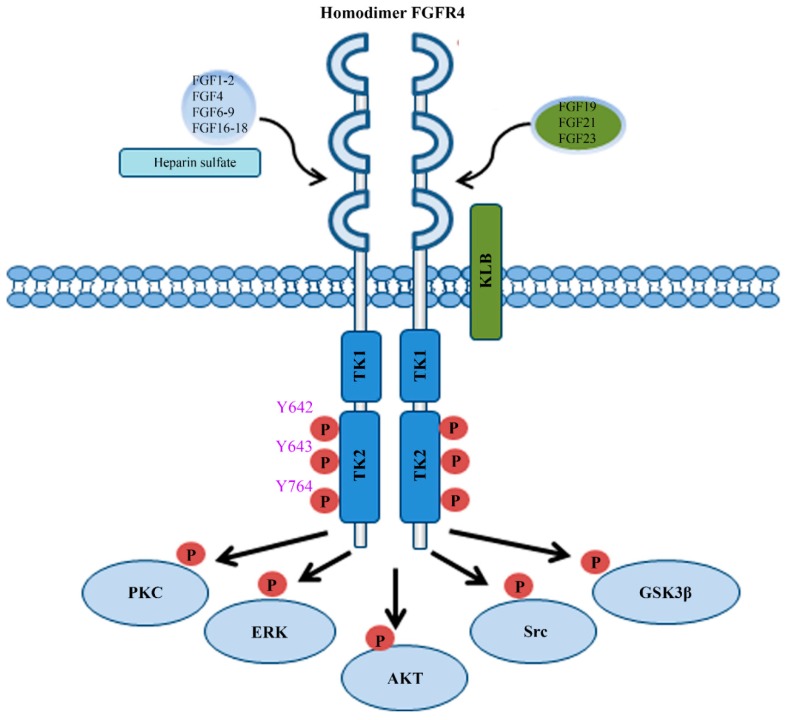
The FGF/FGFR4 signal axis. The signal transduction mediated by the FGF/FGFR4 axis is extremely complex, which includes PKC, ERK1/2, AKT, Src, and GSK3β signaling cascades. The homodimer of FGFR4 forms when binding to either canonical FGF subfamily members (FGF1, FGF2, FGF4, FGF6, FGF7, FGF8, FGF9, FGF16, FGF17, and FGF18) or FGF19 subfamily members (FGF19, FGF21, and FGF23). Heparin or heparin sulfate is required for the binding of canonical FGF subfamily members to FGFR4, whereas KLB acts as a co-receptor of FGFR4 to facilitate FGFR4 interacting with FGF19 subfamily members. When FGFR4 forms protein complexes with FGFs, it can be phosphorylated on three main tyrosine residues: Y642, Y643, and Y764.

FGFR4 is one of four family members harboring tyrosine kinase (TK) domains. The human *FGFR4* gene is located on the long arm of chromosome 5 (5q 35.1). The *FGFR4* gene consists of 18 exons and has five transcript variants with three of them encoding the FGFR4 isoform 1 (Figure 1A) [3]. The 802 amino acid (aa) core region in the FGFR4 protein contains four parts, signal peptide (1–21 aa), extracellular region (22–369 aa), transmembrane region (70–390 aa), and the intracellular region (391–802 aa) (Figure 1B). Similar to the other three FGFR members, the extracellular region of FGFR4 consists of three immunoglobulin-like domains (IgI, IgII, and IgIII), which are essential for specific ligand-binding. IgI is located in 50–107 aa with a length of 97 aa. IgII and IgIII are located in order in 157–241 aa and 264–351 aa. Compared with the other three family members, FGFR4 does not have a splice variant on the IgIII [4]. Several ligand binding sites have been identified, such as 273, 278–280, 309–310, 316, and 337 aa. The TK domains locate in the C terminal from 454–767 aa with several tyrosine (Y) for autocatalysis, such as Y642, Y643, and Y764 (Figure 2).

### 2.2. The Ligands of FGFR4

FGFs are a family of 22 different proteins in vertebrates and are classified into seven subfamilies including FGF1, FGF4, FGF7, FGF8, FGF9, FGF19 ligand subfamily, and FGF11 subfamily [5]. The members of FGF11 subfamily are not ligands of FGFRs and are known as FGF homologous factors [5], while all other six subfamilies work as ligands to bind with FGFR4 (Figure 2) [6]. In other words, ten canonical FGF subfamily members (FGF1, FGF2, FGF4, FGF6, FGF7, FGF8, FGF9, FGF16, FGF17, and FGF18) and three FGF19 subfamily members (FGF19, FGF21, and FGF23) have the potential to bind FGFR4 (Figure 2). Canonical FGFs bind to and activate FGFR4 with heparin/heparin sulfate (HS) [7], while FGF19 subfamily members need β-klotho (KLB) as a co-receptor to bind with FGFR4. FGF1, FGF4, and FGF8, have a higher affinity to bind FGFR4 than other canonical FGFs. Most importantly, FGF19, as an endocrine ligand, has a more specific selective affinity to FGFR4 than other FGFR members [8,9].

### 2.3. The Physiologic Functions of FGFR4

As an important mediator of homeostasis in the liver, FGFR4 function is required for the maintenance of both lipid and glucose metabolism under normal dietary conditions, in addition to its established role in cholesterol [10]. Particularly, FGFR4 activated by endocrine FGF19 represses the gluconeogenesis and stimulates of glycogen and protein synthesis in hepatocytes [11]. The liver-protective effect of FGFR4 becomes even clearer in the model of carbon tetrachloride-induced liver damage, where more significant liver fibrosis was observed in FGFR4 knock-out compared with wild-type (wt) mice [12]. The importance of FGFR4 in controlling bile acids was also established. It has been reported that bile acids secretion and cholesterol metabolism are regulated by FGF19 through binding to FGFR4 in physical activity [13]. It is worth mentioning that the FGF6/FGFR4 pathway plays important role in myoblast differentiation and myotube regeneration [14,15].

## 3. The Genetic Alterations of FGFR4 Gene in Cancer

The high expression levels of *FGFR4* can be detected during fetal human and mouse embryonic development. However, deletion of *FGFR4* does not lead to developmental abnormalities in adult mice only with changed cholesterol metabolism and elevated bile acids [16,17]. The expression of FGFR4 is dramatically decreased although it still consistently expresses in several organs, especially in the liver. Gene alterations of FGFRs, including amplification, translocation, and mutation of gain of function, have been linked to tumorigenesis and cancer progression in solid and hematological malignancies. Recently, one study was conducted to evaluate the alterations of FGFR genes in a variety of cancer types [18], which showed that gene alterations of FGFRs occurred in 7.1% of 4853 solid tumors, with the majority being gene amplification (66% of the aberrations), followed by mutations (26%) and translocations (8%). Amplification was the predominant type of alteration for the *FGFR4* gene, accounting for 78% of all FGFR4 gene alterations. Interestingly, the amplified FGFR4 gene was identified in 10% of breast cancer, which more frequently harbors estrogen- and progesterone-receptor with lymph-node metastases [11]. Unlike FGFR1, the translocation of FGFR4 is very rare in human cancers [18]. Two point mutations in the TK domains of the *FGFR4* gene, K535 and E550, have been identified in rhabdomyosarcoma [19]. Another activating point mutation in *FGFR4* gene (Y367C) inducing constitutive FGFR4 dimerization, has been found in MDA-MB-453 breast cancer cells [20]. Although the gene alteration is relatively low, FGFR4 overexpression has been reported in many types of cancer. Increased FGFR4 mRNA expression has been detected in one-third of hepatocellular carcinoma (HCC) [21]. In another study, elevated FGFR4 mRNA levels were detected in 32% of breast cancer samples [22]. FGFR4 overexpression is also observed in 64% (153/238) of oropharyngeal squamous cell carcinoma and 41% (87/212) of oral squamous cell carcinoma [23]. Overexpressed FGFR4 has also been found in pancreatic carcinomas and derived cell lines, which are mediated by an intronic enhancer activated by hepatic nuclear factor 1 alpha [24]. Additionally, highly FGFR4 expression was detected in rhabdomyosarcoma [19].

As a specific ligand of FGFR4, FGF19 can bind and active FGFR4 with the co-receptor KLB. FGFR4 consistently activated by amplified FGF19 has been identified in several types of cancer. The *FGF19* gene is located on chromosome 11q13.3, a region commonly amplified in human cancer. The amplification of the *FGF19* gene was found in liver cancer, breast cancer, lung cancer, bladder cancer, head and neck squamous cell carcinoma (HNSCC), and esophageal cancer [25,26,27,28,29]. For example, the frequency of the amplified *FGF19* gene is as high as 15% in HCC [26]. Moreover, compared with adjacent normal liver tissues, HCC tissues have significantly elevated mRNA levels of FGF19 [30], suggesting the increased mRNA expression is tightly associated with its amplification. A similar tendency was also identified in HNSCC where FGF19 amplification corresponds with an increased dependency upon FGF19–FGFR4 autocrine signaling [31].

## 4. Mechanisms and Functions of FGFR4 in Cancer Development and Treatment

Accumulating observations indicate that the FGFR4 plays vital roles for cancer development, especially for those harboring *FGF19* amplification. Unlike other family members, the mechanisms and functions of FGFR4 are still poorly characterized at the molecular level in cancer development and progression. Here, the novel observations of mechanisms and functions about oncogenic FGFR4 signaling in cancer development and progression have been summarized and discussed.

### 4.1. FGFR4-Mediated AKT and ERK Signaling Cascades Promote Cancer Development

**Figure 3:**
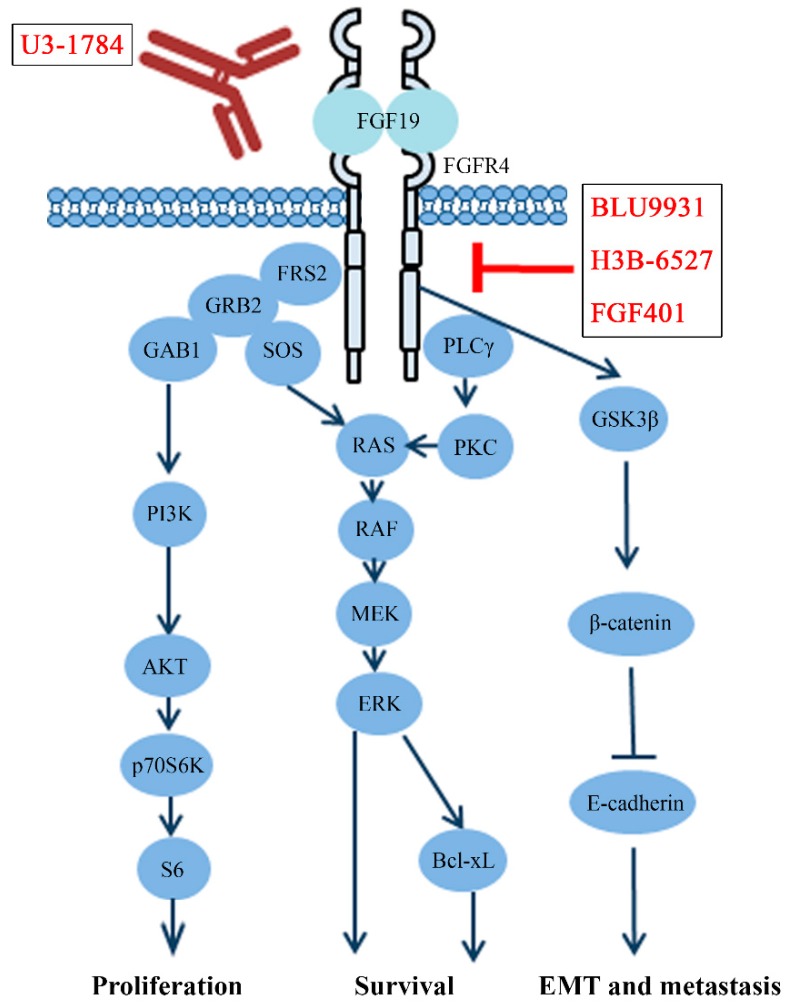
The signal transduction cascades of FGF19/FGFR4 in cancer development and progression. In cancer cells, once FGFR4 receives the extracellular signal from FGF19, it activates many downstream pathways, including PI3K-AKT, MEK-ERK, and GSK3β-β-catenin, leading to increased tumor-promoting activities. FGFR4 activation can be blocked by two non-genetic strategies, using either monoclonal antibodies (e.g., U3-1798) or selective small-molecule inhibitors (e.g., BLU9931, H3B-6527 and FGF401).

The MAPK-ERK and PI3K-AKT signaling are two main pathways regulated by the FGF/FGFR protein complexes. After binding FGFs with HS or KLB, FGFR4 will be activated through autophosphorylation and forms a homodimer (Figure 2). FGFR4 also has the potential to form a heterodimer receptor with other family members, especially with FGFR3 [32,33]. Mechanistic studies showed that phosphorylated FGFR4 recruits and phosphorylates two important intracellular targets, phospholipase γ (PLCγ) and FGFR substrate 2 (FRS2) [4]. MAPK then can be stimulated by activated protein kinase C (PKC) through PLCγ. Meanwhile, the MAPK and PI3K-AKT pathway can be triggered by activated FRS2 through recruitment of growth factor receptor bound 2 (GRB2) (Figure 3) [4]. Upregulated activity of AKT and ERK1/2 leads to enhanced cell proliferation and survival in HCC upon the activation of FGF19/FGFR4 signaling (Figure 3) [34,35,36].

*FGF19* is more highly expressed in the breast cancer tissue than the adjacent normal tissue [37], and co-expression of *FGFR4* and *FGF19* accounts over 28% primary breast cancer [38]. AKT phosphorylation is strongly associated with co-expression of *FGF19* and *FGFR4*, which can be blocked with FGF19 antibody (1A6) or siRNA-mediated silencing of *FGF19* in breast cancer cells [27]. Our recent findings reveal that FGFR4-mediated hyperactivation of AKT increases breast cancer cells proliferation, but not metastasis [37]. Inactivation of FGFR4 by its inhibitor BLU9931 significantly attenuates FGF19-induced tumor-promoting activity, suggesting interruption of FGFR4 function is sufficient to affect FGF19-driven breast cancer [37].

Our recent study demonstrates that *FGF19* amplification and overexpression are associated with a poorer overall survival rate for HNSCC patients, provoking FGFR4-dependent ERK/AKT-p70S6K-S6 signaling activation to increase HNSCC cell proliferation [31]. Blocking activation of FGFR4 by small hairpin RNAs (shRNAs) or BLU9931, not only attenuates FGF19-induced ERK1/2 and AKT activation, but also abrogates its ability to induce cell proliferation [31]. FGFR4-induced activation of ERK1/2 and AKT pathways was also correlated with increased cell proliferation and survival in colorectal cancer (CRC) [39].

### 4.2. The FGF19–FGFR4 Axis Promotes Epithelial-Mesenchymal Transition (EMT) to Accelerate Metastasis

The FGF19–FGFR4 axis has been linked to metastasis and poor survival [26]. FGFR4 is predominantly expressed in the liver and responsive for FGF19 stimulation to regulate cholesterol metabolism. There is no doubt that elevated FGF19–FGFR4 signaling is associated with HCC progression, especially for metastasis [26,40]. Our research team has demonstrated that the FGF19/FGFR4 axis facilitates HCC cell EMT through upregulating GSK3β-β-catenin signaling and consequently increases HCC metastasis (Figure 3) [30]. Recently, a vital role of FGFR4 was found in CRC metastasis. Activated FGFR4 phosphorylates AKT, ERK1/2, and Src, leading to increased CRC cell invasion. Silencing FGFR4 reduces adhesion, migration, and invasion of CRC cells [39]. Further study shows that depletion FGFR4 by CRISPR-Cas9 results in the morphological changes and reduced metastasis ability, accompanied by upregulation of E-cadherin and downregulation of Snail and other EMT mediators [39]. Moreover, FGFR4-GSK3β-β-catenin is also elucidated in CRC metastasis. Elevated expression of *Forkhead box C1* (*FOXC1*) is tightly correlated with metastasis of CRC, and FGFR4 is the main target of this gene [41]. BLU9931, the specific inhibitor of FGFR4, can inhibit the activation of GSK3β/β-catenin induced by *FOXC1* overexpression in vitro and metastatic colonization of CRC in vivo [41].

### 4.3. FGFR4-Associated Chemotherapy Resistance in Cancer

Cancer cells may develop a mechanism that inactivates the drug which represents the main obstacle for cancer treatment. It can be achieved by cancer cells through different mechanisms, such as drug inactivation, drug target alteration, drug efflux, DNA damage repair, cell death inhibition, and EMT [42]. The FGF19–FGFR4 axis has participated in chemotherapy resistance in several types of cancers. The expression levels of FGFR4 are significantly increased in doxorubicin-resistant breast cancer clones [43]. Moreover, FGFR4 overexpression has been detected in those insensitive breast cancer cell lines to doxorubicin [43]. Silencing *FGFR4* with small interfering RNA (siRNA) in chemo-resistant clones increases their sensitivity to doxorubicin. Furthermore, inhibition of FGFR4 with an antagonistic antibody also enhances the sensitivity of endogenously FGFR4-expressing cell lines to doxorubicin. Inhibition of apoptosis by FGFR4 is the main mechanism of doxorubicin resistance in breast cancer [43]. Bcl-xL, an anti-apoptotic protein, is upregulated by FGFR4 via MAPK cascade and responsive for the increased resistance to doxorubicin [43]. Other studies indicate that upregulation of FGF19–FGFR4 signaling increases drug resistance to doxorubicin in basal-like breast cancer [27]. Inactivation of FGFR4 signaling by an anti-FGF19 antibody or siRNA-mediated *FGF19* gene silencing, can sensitize FGFR4^+^/FGF19^+^ breast cancer cells to doxorubicin treatment [27]. Increased sensitivity to 5-fluorouracil (5-FU) or oxaliplatin treatment has also been observed after *FGFR4* silencing in CRC cells [44].

As a multiple TKI, sorafenib is an efficient target therapy agent to treat HCC. However, sorafenib is always restricted to continuous administration by occurring drug resistance with unknown mechanisms [45,46]. Recently, our study shows that activation of the FGF19–FGFR4 axis is one of the main mechanisms for sorafenib resistance in the treatment of HCC [47]. The outbalanced oxidative stress induced by reactive oxygen species (ROS) plays a pivotal role in apoptosis [48]. Mechanistically, sorafenib induces ROS-associated apoptosis, but this can be suppressed by *FGF19* overexpression in HCC cells. The FGF19–FGFR4 axis has the potential to assist HCC cancer cells to escape apoptosis in sorafenib treatment through suppression of ROS. *FGFR4* knockout increases the sensitivity to sorafenib treatment in HCC cells, accompanied by enhanced cell apoptosis [47]. Silencing the *FGF19* gene or inactivating FGFR4 with the FGFR-pan inhibitor ponatinib, significantly increases the sensitivity to sorafenib in sorafenib-resistant HCC cells, with induced apoptosis and accumulated ROS generation [47]. Additionally, a similar phenomenon is also observed in young adult mouse colonic epithelial cells. Upregulating FGF19–FGFR4 signaling significantly reduces ROS-mediated apoptosis caused by H_2_O_2_ through blocking the caspase-3 pathway [49], which also prevents prostate cancer cells from apoptosis in TNFα treatment [50].

## 5. Develop Specific FGFR4 Inhibitors Targeting Cancer Harboring Elevated FGF19/FGFR4 Signaling

As a promising target, FGFR4 attracts intensive pharmaceutical and academic attention to develop novel target therapy against cancers driven by FGFR4. Three strategies have been developed to target FGFR4, including neutral antibodies, antisense oligonucleotides, and small molecule inhibitors. Two monoclonal neutralizing antibodies of FGFR4, LD-1, and U3-1784, have been developed to competitively targeting extracellular Ig domains of FGFR4. The therapeutic efficacy of U3-1784 is currently being evaluated in Phase I clinical trials for the treatment of HCC and other advanced solid tumors [51] (Table 1 and Figure 3). As an antisense oligonucleotide targeting FGFR4 mRNA, ISIS-FGFR4RX has entered a Phase I clinical trial for obesity (NCT02476019). However, the potential anticancer activity of ISIS-FGFR4RX has not been reported.

Comparing two strategies above, targeting FGFR4 using small-molecule inhibitors is more feasible and can be developed through structure-guided drug design. Not surprisingly, multi-targeted tyrosine kinase inhibitors (mTKIs) can be used to inactivate FGFR4 by disrupting ATP binding in its TK domains. The anticancer activity of many mTKIs, including lenvatinib and ponatinib, have been tested on FGFR-driven solid tumors in animals or in clinical trials (Table 1) [52,53,54]. However, the limited selective activity of mTKIs on FGFRs induces less efficiency and increases side effects in these treatments. Therefore, pan-FGFR inhibitors are developed and are being evaluated in clinical trials to treat cancers driven by abnormal FGFR pathways. Most of these inhibitors target ATP binding pocket in the TK domains of FGFRs through reversible or covalent bonds. For example, ponatinib can impede the autophosphorylation activity of FGFRs by binding to the hinge region of FGFRs and block the ATP-binding cassette motif [5]. As such, ponatinib has the great potential to inhibit the enzyme activity of FGFRs which are always hyperactive in cancer cells. Other inhibitors in this category include ATP-competitive inhibitors NVP-BGJ398 [55] and AZD4547 [56], ATP-binding pocket inhibitor LY2874455 [57], and FGFRs-FIIN-3 which generates a covalent bond with a conserved cysteine located in the ATP binding site (Table 1) [58]. However, the low specificity of these pan-FGFR inhibitors to FGFR4 cannot sufficiently suppress the oncogenic FGFR4 signaling. For example, the IC_50_ of AZD4547 on FGFR4 is over 100-fold higher than other FGFR members [59]. Moreover, inevitable on-target toxicities and off-target activity resulting from the use of nonspecific FGFR inhibitors lead to several adverse effects such as soft-tissue mineralization and hyperphosphatemia [60]. Such disadvantages eventually limit their usage in cancer patients.

Compared with other FGFR family members, FGFR4 is more specifically expressed in the liver and several other organs for bile acid secretion and cholesterol metabolism. Therefore, the generation of more specific inhibitors which only abolish FGFR4 can improve FGFR4 sensitivity and overcome the drawbacks of pan-FGFR inhibitors. BLU9931 is the first selective FGFR4 inhibitor for the treatment of HCC with an activated FGFR4 signaling pathway [61,62]. As a novel irreversible kinase inhibitor, BLU9931 creates a covalent bond with Cysteine 552 near the ATP-binding site that is only present in FGFR4 among FGFRs [62]. BLU9931 can effectively inhibit HCC tumor harboring elevated FGF19–FGFR4 axis in vivo. Moreover, BLU9931 also displays the potent anticancer ability in breast cancer, CRC, and HNSCC with upregulated FGF19–FGFR4 signaling [37,41]. BLU554 was derived from BLU9931 with improved pharmaceutical properties, which is now in Phase I clinical trial to treat HCC with elevated FGF19–FGFR4 axis (NCT02508467) (Table 1) [63]. H3B-6527 is another selective FGFR4 inhibitor currently in clinical trials for HCC treatment (Table 1). H3B-6527 also targets Cysteine 552 through forming a covalent bond near the ATP binding site of FGFR4, and it exhibits an inhibitory effect on FGFR4 activation in FGF19-driven HCC in vitro and in vivo [64]. By studying a panel of 40 HCC cell lines and 30 HCC patient-derived xenograft models, the expression levels of FGF19 are implicated as a predictive biomarker for H3B-6527 response [64]. Moreover, the combination of H3B-6527 with the CDK4/6 inhibitor palbociclib has a superior effect on the repression of tumors in a xenograft model of HCC [64]. FGF401 is another selective FGFR4 inhibitor, which is under investigation in a phase I/II study to treat HCC with FGFR4 and KLB expression (NCT02325739) [65] (Table 1). FGF401 is evaluated to treat HCC as the single use or combined with a humanized anti-PD1 IgG4 antibody PDR001 [65]. These novel FGFR4-targeting therapies provide a novel and promising approach which could potentially be developed into a therapeutic strategy to combat cancer.

## 6. Conclusions

Increasing evidence indicates that upregulation of FGF19–FGFR4 signaling plays an essential role in tumorigenesis and cancer progression. FGFR4 has been proven as an attracted target to develop a novel therapy for the subgroup of cancers associated with the FGF19–FGFR4 pathway. Given that overexpression of FGFR4 significantly correlates with EpCAM, a marker of hepatic cancer stem cells, within the fatty liver-steatosis-cirrhosis-HCC sequence [66], FGFR4 may have the ability to regulate cancer stem cells and lead to chemoresistance in HCC or other cancers. Gaining these insights will improve our comprehensive understanding of the role of FGFR4 in cancer development and treatment. Recently, more specific inhibitors targeting FGFR4 have been developed and evaluated, which are demonstrating promise as a single agent therapy or in combination with other anticancer agents. Thus, there is no doubt that FGFR4-targeting inhibitors offer the most immediate prospects of reducing cancer mortality rate. Perhaps, we can design stapled peptides [67] to incorporate the hydrophobic staple at the interface of FGF19–FGFR4 binding sites, which would increase the specificity of signal targeting compared to the FGFR4 inhibitors that are commercially available. Although the critical role of FGFR4 in metastasis has been demonstrated in animal models of several cancers, prospective studies are warranted to provide evidence regarding the therapeutic efficacy of FGFR4 inhibitors in clinical metastatic cancers.

## Abbreviation

aaamino acidCRCcolorectal cancerEpCAMEpithelial cell adhesion moleculeFGFfibroblast growth factorFGFRfibroblast growth factor receptorFOXC1Forkhead box C1FRS2FGFR substrate 2GRB2growth factor receptor bound 2HCChepatocellular carcinomaHNSCChead and neck squamous cell carcinomaHSheparin sulfatemTKImulti-targeted tyrosine kinase inhibitorPLCγphospholipase γ
*p70S6K*
p70 ribosomal protein S6 kinaseROSreactive oxygen speciesshRNAsmall hairpin RNAsiRNAsmall interfering RNATKtyrosine kinase

## Figures and Tables

**Table 1:** Clinical trials of FGFR4 inhibitors for cancer treatment.

Drug	Structure	Target(s)	Cancer Type	Clinical Trial Number/Phase
Ponatinib	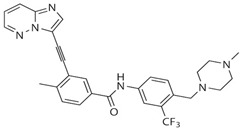	Multiple RTKs, including FGFRs	Advanced solid tumors with activating mutations of FGFRs	NCT02272998/II
AZD4547	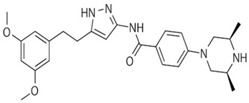	Pan-FGFRs	Recurrent malignant glioma expressing FGFR-TACC gene fusion	NCT02824133/I/II
LY2874455	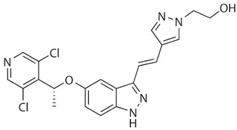	Pan-FGFRs	Advanced cancer	NCT01212107/I
NVP-BGJ398	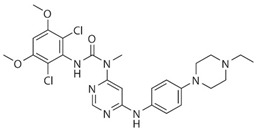	Pan-FGFRs	Solid tumors and hematologic malignancies with FGFR genetic alterations	NCT02160041/II
BLU554	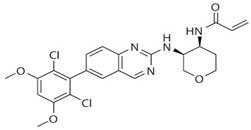	FGFR4	Hepatocellular carcinoma	NCT02508467/I
FGF401	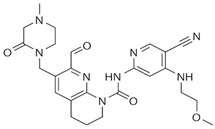	FGFR4	Hepatocellular carcinoma and other solid tumors	NCT02325739/I/II
H3B-6527	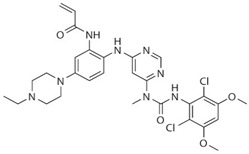	FGFR4	Advanced hepatocellular carcinoma and intrahepatic cholangiocarcinoma	NCT02834780/I
U3-1784	Monoclonal antibody	FGFR4	Hepatocellular cancer and other advanced solid tumors	NCT02690350/I
